# Extrinsic Effects on the Optical Properties of Surface Color Defects Generated in Hexagonal Boron Nitride Nanosheets

**DOI:** 10.1021/acsami.1c11060

**Published:** 2021-09-14

**Authors:** Marie Krečmarová, Rodolfo Canet-Albiach, Hamid Pashaei-Adl, Setatira Gorji, Guillermo Muñoz-Matutano, Miloš Nesládek, Juan P. Martínez-Pastor, Juan F. Sánchez-Royo

**Affiliations:** † Instituto de Ciencia de Materiales, Universidad de Valencia (ICMUV), P.O. Box 22085, 46071 Valencia, Spain; ‡ Institute for Materials Research, Material Physics Division University of Hasselt, Wetenschapspark 1, B 3590 Diepenbeek, Belgium

**Keywords:** hexagonal boron nitride, 2D materials, color defects, photoluminescence, interfaces

## Abstract

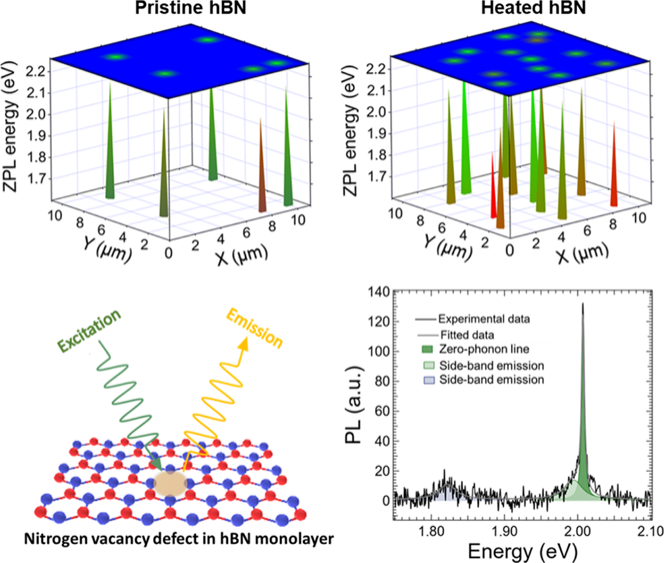

Hexagonal boron nitride (hBN) is a wide-band gap van der Waals material able to host light-emitting centers behaving as single photon sources. Here, we report the generation of color defects in hBN nanosheets dispersed on different kinds of substrates by thermal treatment processes. The optical properties of these defects have been studied using microspectroscopy techniques and far-field simulations of their light emission. Using these techniques, we have found that subsequent ozone treatments of the deposited hBN nanosheets improve the optical emission properties of created defects, as revealed by their zero-phonon linewidth narrowing and reduction of background emission. Microlocalized color defects deposited on dielectric substrates show bright (≈1 MHz) and stable room-temperature light emission with zero-phonon line peak energy varying from 1.56 to 2.27 eV, being the most probable value 2.16 eV. In addition to this, we have observed a substrate dependence of the optical performance of the generated color defects. The energy range of the emitters prepared on gold substrates is strongly reduced, as compared to that observed in dielectric substrates or even alumina. We attribute this effect to the quenching of low-energy color defects (these of energies lower than 1.9 eV) when gold substrates are used, which reveals the surface nature of the defects created in hBN nanosheets. Results described here are important for future quantum light experiments and their integration in photonic chips.

## 1 Introduction

Hexagonal boron nitride (hBN) is a low-dimensional van der Waals material with a honey-comb crystal lattice structure similar to graphene (see Figure S1a in Supporting Information—SI). It is an electrical insulator with a large band gap of ≈6 eV^1^ and an exciton binding energy of ≈130 meV.^2^ The low-dimensional character, smooth surface, insulating properties, and very high chemical stability^3^ make hBN a suitable ideal platform for the fabrication of enhanced electronic and optoelectronic nanodevices, where hBN can be used as a protective and dielectric layer.^4−6^ Another outstanding property of hBN is related to recently discovered point color defects, which are very attractive for a variety of applications as single-photon sources.^7−11^ The physical origin of these point color defects is not yet well-known, it is related to the presence of several different crystal vacancies^12−14^^,^ and atom impurities^13,15−17^ in the hBN crystal lattice. They lead to very bright^8,10,18−20^ and stable^8,11^ light emission at visible wavelengths operating from low temperature (≈10–20 K)^9,21^ to high temperature up to ≈1073 K.^22^ Interestingly, some pieces of evidence appear to point out optically addressable spin-dependent properties of some color defects.^23^

These color defects can be created by high energy electron^8,24^ or oxygen^25^ irradiation, pulsed laser excitation,^26^ thermal annealing,^8,9,27^ plasma treatment,^9,27^ or directly during CVD growth.^28,29^ The structural quality of the hBN crystal lattice seems to play an important role in luminescent emission properties of the color defects. Especially, lattice imperfections might lead to electron-phonon coupling and the formation of undesired phonon side-band (SB) emission.^30,31^ Furthermore, extrinsic surface defects might serve as charge traps.

**Figure 1:**
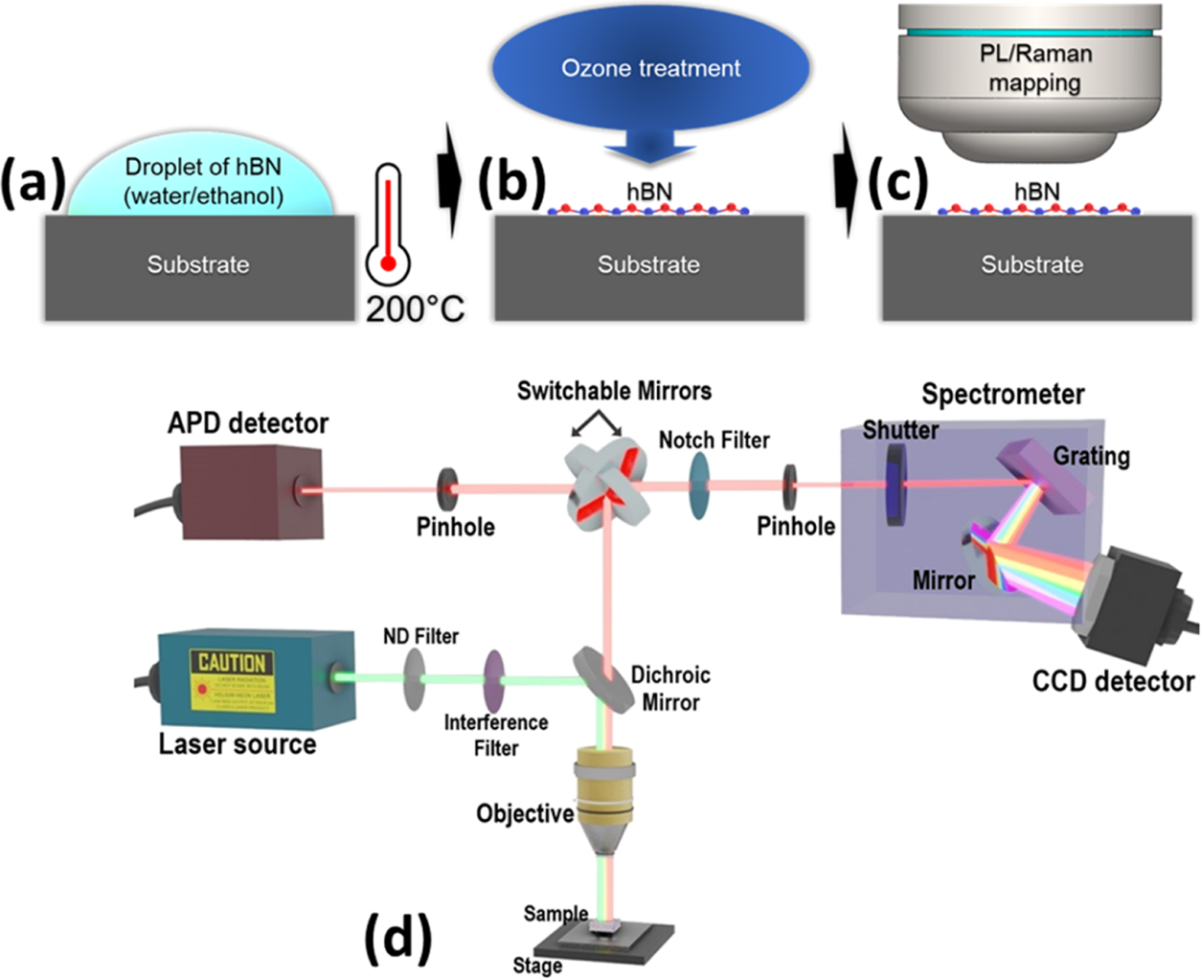
Preparation of light-emitting nanosheets including (a) drop-casting of water/ethanol hBN solution with a concentration of 5.4 mg/L and a volume of 10–20 μL on the heated substrate up to 200 °C in order to evaporate organics and enhance the presence of light emitters in the crystal lattice followed by (b) ozone post-treatment to remove organic residues and improve emission properties. Optical properties of light emitters were then investigated by (c) micro-PL and Raman mapping. (d) Scheme of the confocal microspectroscopy system. Micro-Raman spectroscopy was used to determine the hBN thickness and micro-PL spectroscopy was used to study light emission of atomic color defects in the hBN lattice. A green excitation laser beam with a wavelength of 532 nm was focused through a pinhole to the focal plane of the hBN crystal lattice to achieve microlocalization. Raman spectra corresponding to the hBN lattice vibrations and PL spectra from microlocalized color defects were detected using a CCD camera. PL maps were recorded using an APD.

In this work, we have generated color defects in hBN nanosheets deposited on dielectric and metallic substrates by thermal treatment processes. To better understand light emission properties of these color emitters and clarify their physical origin, we have applied microspectroscopy techniques (see Figure 1d) and far-field angle-dependent simulations of the in-plane^30,32,33^ photoluminescent (PL) emission. Using these techniques, we have found an enhancement of the density of color defects in the dispersed nanosheets and a significant improvement of the crystal hBN lattice quality, after thermal treatment. Above that, subsequent ozone treatment results in narrowing of the zero-phonon linewidth (ZPL) of the emitter and reduction of its background emission. ZPL linewidth and contribution of SB emission in hBN monolayers and thicker nanosheets are comparable, indicating a good surface quality with a significantly lower occurrence of charge traps and defects, especially due to the used ozone surface post-treatment. In addition to these findings, we have studied the influence of the substrate nature on the optical properties of the emitters. The generated color defects deposited on dielectric and oxidized metallic Al substrates emit in a very broad energy range from 1.56 to 2.27 eV with the most probable value at 2.16 eV. However, for metallic Au substrates, the range of ZPL energy is strongly reduced from 1.91 to 2.21 eV, that is, color defects emitting at lower energies are quenched. The observed quenching of the low-energy emitters of hBN nanosheets deposited on Au substrates suggests that color defects are localized preferably on the surface. In addition, we have found both experimentally and theoretically that a maximum PL emission is collected from hBN nanosheets deposited on the Si substrate. On the other hand, some PL losses have been found on the SiO_2_/Si substrate and metal substrates.

## 2 Materials and Methods

### 2.1 Preparation Method

Few-layer hBN nanosheets with a lateral size of 50–200 nm and a thickness of 1–5 monolayers were dispersed using a pristine hBN water/ethanol solution (5.4 mg/L) from Graphene Supermarket by drop-casting (10–20 μL) onto heated (200 °C) clean substrates in air (see Figure 1a,b) for 5 min. Substrate heating was used to enhance the occurrence of atomic color defects in the hBN crystal lattice and to remove eventual organic residues from the hBN surface.^34,35^ After that, we applied ozone surface treatment of hBN nanosheets using the Ossila UV ozone cleaner (room temperature, 10 min) in order to improve the quality of their light emission.^27,35^ Four different substrates were used: SiO_2_/Si (SiO_2_ thickness of 285 nm), Si (Si thickness of 380 μm), Al/SiO_2_/Si (Al thickness of 100 nm), and Au/SiO_2_/Si (Au thickness of 30 nm).

### 2.2 Spectroscopy Techniques

We used two microspectroscopy techniques to study the optical properties of the color defects. The scheme of micro-Raman and micro-PL techniques is depicted in Figure 1d. Micro-Raman and micro-PL spectroscopies were carried out using a confocal Raman microscope Horiba-MTB Xplora at room temperature using a green laser illumination with 532 nm excitation wavelength, 2.1 mW laser power, and 100× objective (N.A. = 0.9, WD = 0.21 mm). The thickness of hBN nanosheets was determined using micro-Raman spectroscopy from peak shift of the E_2g_ phonon mode.^36^ Raman and PL spectra were taken at the same laser spot. PL maps with the corresponding PL spectra taken at localized point defects were carried out at room temperature using a home-built confocal microscope with green (532 nm) CW Gem laser from Laser Quantum, 1 mW laser power, 100× objective (N.A. = 0.8, WD = 3.4 mm), and single-photon silicon avalanche photodiode (APD) from Excelitas for PL mapping and, finally, we used a monochromator combined with a charge-coupled device (CCD) camera for conventional PL spectra recording.

### 2.3 Simulations

The far-field radiation patterns of the structures were calculated in 2D COMSOL Multiphysics, frequency domain, RF module, using the Lorentz reciprocity theorem,^37−39^ based on the finite element method (FEM). The angular reflectance spectra of the aforementioned structures have been calculated using well-known transfer matrix methods (TMMs).^40−43^ The calculation method is explained in more detail in Supporting Information. Band bending of electron energy levels for Au, Al_2_O_3_, and SiO_2_ substrates was simulated in AMPS-1D software (Analysis of Microelectronic and Photonic Structures) using numerical solutions of the Poisson’s equation. For the simulation, we used the following parameters: an hBN band gap of 5.76 eV,^9^ an hBN electron affinity of 1 eV,^44^ and a Au work function of 5.5 eV.

### 2.4 X-ray Photoemission (XPS)

Measurements were performed in a SPECS GmbH system (base pressure 1.0 × 10^–10^ mbar) equipped with a PHOIBOS 150 2D-CMOS hemispherical analyzer. Photoelectrons were excited with the Al-K_a_ line (1486.7 eV) of a monochromatic X-ray source μ-FOCUS 500 (SPECS GmbH). Measurements were taken at room temperature with a pass energy of 20 eV.

## 3 Results

### 3.1 Preparation of Light-Emitting hBN Nanosheets

The hBN nanosheets with a thickness of 1–5 monolayers (L) were prepared by drop-casting their colloidal solution on different substrates (see Figure 1a,b). Optical microscopy and scanning electron microscopy (SEM) images of dispersed hBN nanosheets on the Si substrate are depicted in Figure S1b–e in Supporting Information. The substrates were heated in order to fastly evaporate organic residues and increase the presence of atomic color defects in the crystal lattice. As a final step, the dispersed nanosheets were exposed to ozone surface treatment to enhance PL emission properties.^35^

**Figure 2:**
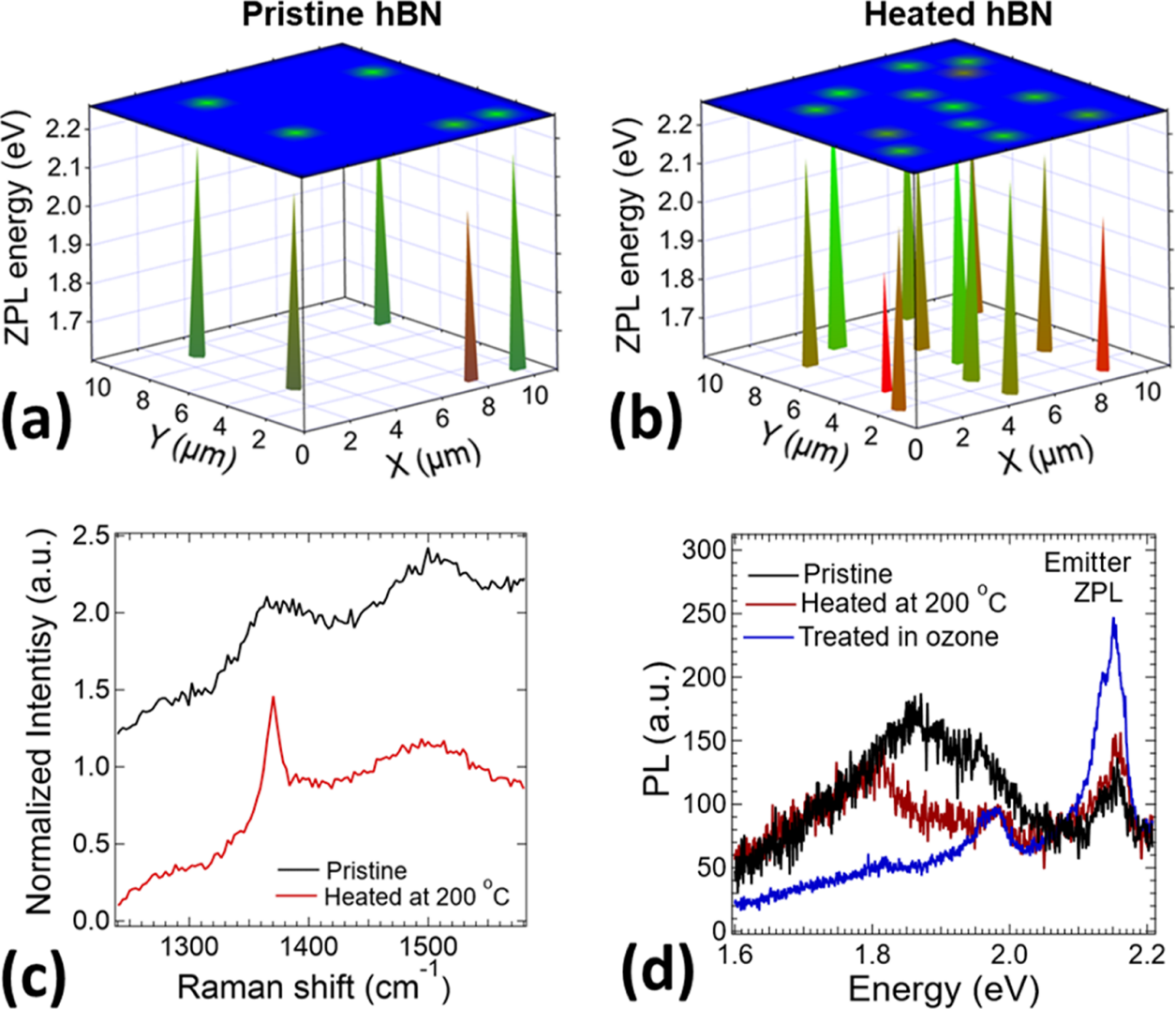
Photoluminescence (PL) map of microlocalized light emitters in (a) pristine hBN nanosheets dispersed on a Si substrate without applied heating and in (b) Si substrate subsequently heated at 200 °C for some minutes, the latter showing more than 2 times higher amount of emitting color defects. (c) Comparison of Raman spectra for pristine hBN and heated hBN showing an improvement of crystal quality by heating. (d) PL spectra comparison of a microlocalized light emitter in pristine hBN dispersed on the Si substrate, after its post-heating at 200 °C in air and ozone treatment showing a high improvement of the light emission and reduction of unwanted PL background especially with the ozone post-treatment.

In order to demonstrate an improvement of the light emission properties by the used preparation technique, we monitored a microlocalized light emission in pristine and treated hBN by PL and Raman spectroscopy. In our investigation, we found more than 2 times higher occurrence of color defects in hBN nanosheets deposited on the heated Si substrate than in pristine nanosheets dried at room temperature, by comparing their respective micro-PL maps (see Figure 2a,b). We also used Raman spectroscopy to investigate the crystallinity of the pristine and heated nanosheets (Figure 2c). Raman spectra show a broad Raman peak for pristine hBN and a sharp Raman peak centered at 1370.3 cm^–1^ for heated hBN, suggesting a noticeable improvement of the crystal quality which reduces the electron–phonon interaction affecting the light emission properties of color defects.

Figure 2d shows the PL spectra measured in light emitters localized in pristine and treated hBN by heating and ozone treatment. Comparing these three PL spectra, it is clearly visible that the post-treatment, especially by ozone, highly improves light emission properties leading to narrowing of the ZPL emission linewidth together with increased PL intensity and reduction of unwanted PL background and hence enhancing the optical quality of the transition. Note that, the unwanted PL background around 1.8 eV was detected in all pristine hBN nanosheets and also on the bare substrate, which disappeared after the post-treatment. These results suggest that the origin of the PL background can be attributable to organic residues.

### 3.2 Phonon Interactions with Color Centers

**Figure 3:**
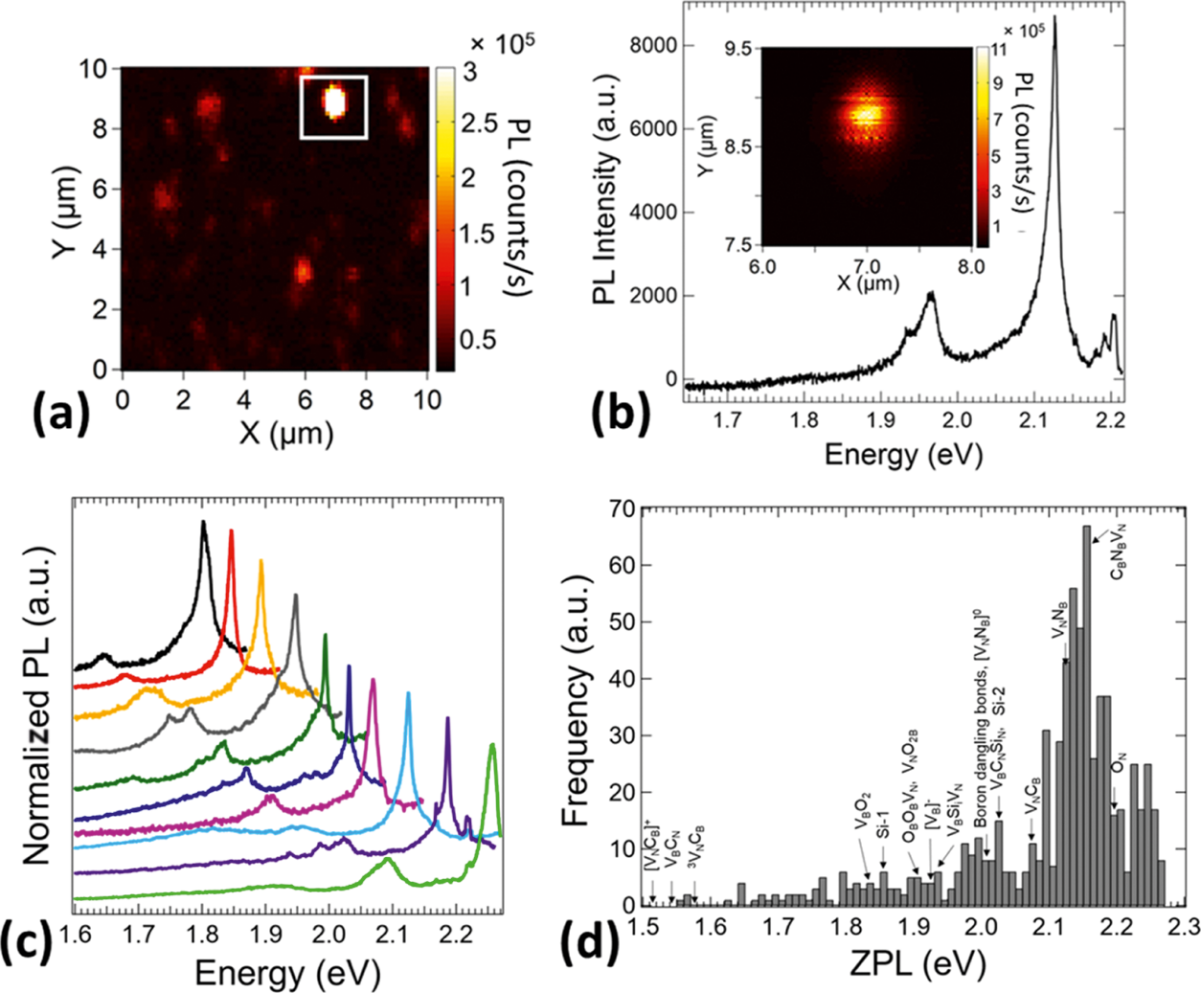
(a) Photoluminescence (PL) map of microlocalized emission from emitters in hBN nanosheets. The white highlighted area represents the brightest point defect emission. (b) PL map corresponding to the zoomed image of the highlighted point defect (inset) in (a) with the corresponding measured PL spectra. (c) PL spectra of zero-phonon line (ZPL) and phonon side-band (SB) emission across the visible spectrum. PL intensity is normalized to the maximum intensity value and shifted on the *Y*-axis for a better visualization. (d) Histogram of monitored broad ZPL energies on all substrates used in this work. We have labeled a possible origin of the impurities and vacancies responsible for the emitters found, according to the literature.^9,13,15,47,48^ The most probable emitter seems to lie at 2.16 eV.

A PL map (10 × 10 μm) of microlocalized point emission measured in a region of the sample partly covered by multiple hBN nanosheets is shown in Figure 3a. Figure 3b shows a zoomed image (2 × 2 μm) of the brightest emitting point (inset) and its corresponding PL spectrum with ZPL at 2.13 eV and also the presence of phonon SB replicas around 1.95 eV. We have studied PL lineshapes and intensities of various point defects with ZPL transition energies centered across the visible spectrum region and similar ZPL and phonon SB distribution (Figure 3c) as reported in the literature.^8,30^ Many of the light emission properties are highly determined by the crystal quality, especially the presence of stacking fault, dislocations,^45^ surface charges,^29^ and defects affecting phonon interactions,^31^ ZPL shift,^45^ and lifetime.^46^ Therefore, the hBN fabrication process along with the technique for the creation of color defects and post-treatments plays an important role in enhancing the quality of the light emission.

Figure 3d shows the histogram of recorded ZPL central emission of all measured hBN nanosheets varying from 1.56 to 2.27 eV with maxima at 2.16 eV, which is consistent with results repored in the literature.^28,46^ The wide range of emission energies denotes the existence of different types of color defects. However, their origin is still not clear. Possible candidates are boron and nitrogen crystal vacancies^12−14,18^ and atom impurities^13,15−17^ including oxygen, carbon, silicon, and hydrogen localized within all crystal volume,^24^ or dangling bonds,^47^ typically localized near crystal edges or grain boundaries. They typically emit light across their band gap with a wide range of transition energies from ultraviolet^14^ to visible and near-infrared^8,11^ spectrum. One can argue that the existence of a large variety of multiple defect types with different crystal vacancies^12−14,18^ and impurities^13,15−17^ should be an important factor to be taken into account. We compared reported multiple atomic defects^13,15,47,48^ with calculated ZPL central emission energies to ZPL central emission energies obtained in this work (see Figure 3d); however, there are still many unknown ZPL central energy emission across the visible spectrum. Another possible reason for the large emission energy range could be based on extrinsic factors as lattice strain,^19,20,49^ temperature,^22,50^ or Stark shift.^51−53^

### 3.3 Optical Emission Analysis as a Function of Substrate Material

**Figure 4:**
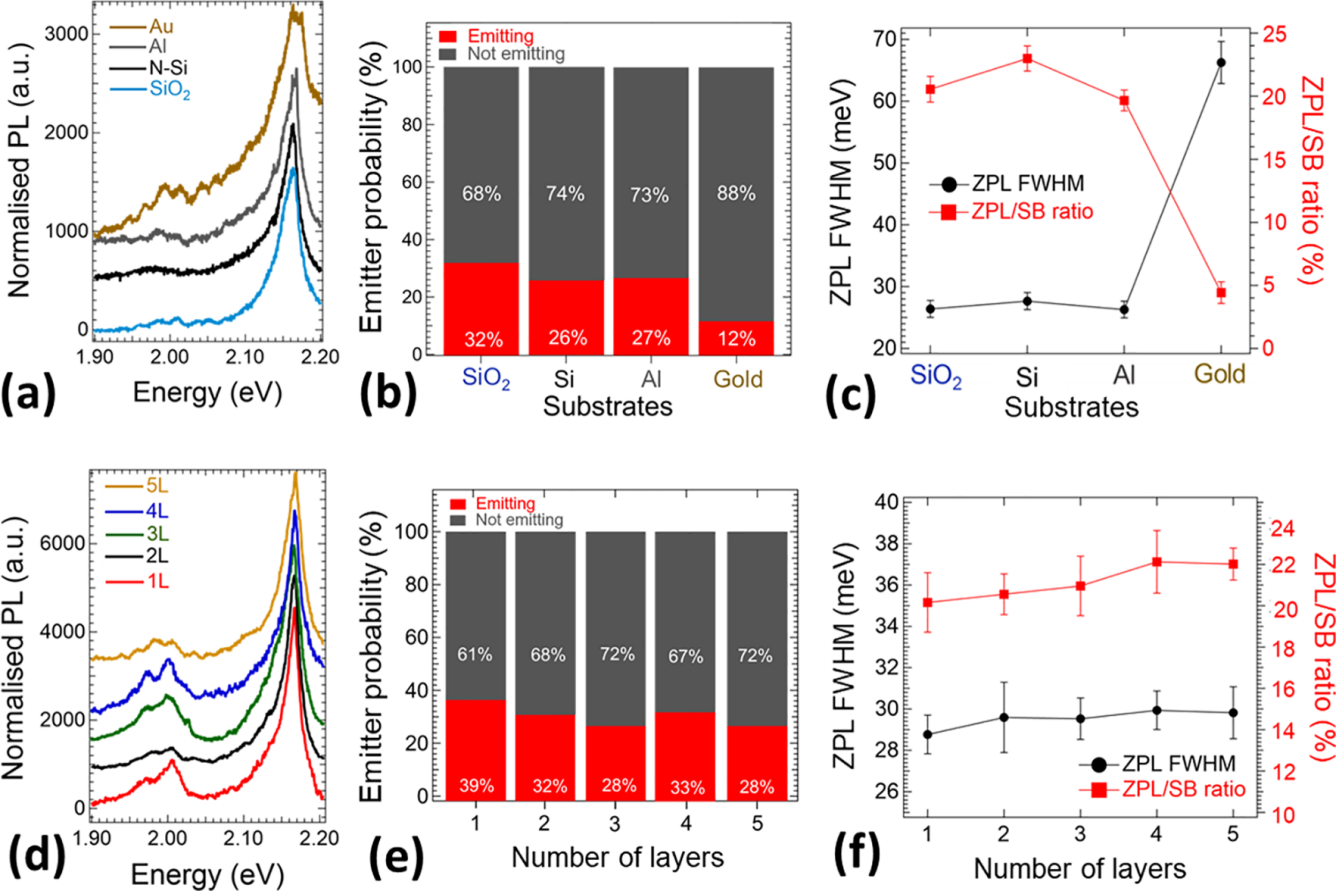
(a) Normalized photoluminescence (PL) spectra of hBN nanosheets dispersed on Au, Al, Si, and SiO_2_ substrates. Spectra are normalized to the maximum PL intensity and shifted on the y-axis for a better visualization. (b) Probability of finding an emitter per 1 μm2 surface of hBN nanosheets, as calculated by counting the number of 1 μm^2^-surface areas of hBN with and without emitters. (c) Average full width at half maximum (fwhm) of the zero-phonon line (ZPL) peaks and average ZPL/side-band (SB) intensity ratio of color defects observed in hBN nanosheets dispersed on the different substrates used in this work. The fwhm and ZPL/SB ratio were calculated by Lorentzian peak fitting. Error bars represent the standard deviation. (d) Normalized PL spectra of hBN nanosheets of thickness ranging from 1 layer (L) to 5 L dispersed on Si substrates. Spectra are normalized to the maximum PL intensity and shifted on the *Y*-axis for a better visualization. (e) Probability of finding an emitter per 1 μm2 surface of hBN nanosheets dispersed on Si substrates, as a function of thickness of the nanosheet. (f) Average fwhm of the ZPL peaks and average ZPL/SB intensity ratio of color defects observed in hBN nanosheets of different thickness dispersed on Si substrates. The fwhm and ZPL/SB ratio were calculated by Lorentzian peak fitting. Error bars represent the standard deviation.

First, we investigated the light emission of hBN nanosheets deposited on different substrates, these being not only dielectric ones (SiO_2_ or Si) but also metals (Al and Au). Figure 4a shows an example of PL spectra recorded on Au, Al, Si, and SiO_2_ substrates with a ZPL energy around 2.16 eV and high similarity except for Au with a bit broader peak and PL background. We calculated a probability of finding an emitter in hBN nanosheets deposited on different substrates using PL and Raman mapping. The emitter probability (the amount of emitting and nonemitting ones, with a constant Raman intensity signal in hBN nanosheets) on different substrates is presented in Figure 4b. Note that, color defects in nonemission states might be also present. All substrates show a relatively high emitter probability ≈30% except for the thin Au metallic layer with a reduced emitter probability to 12%. The relatively high probability of created emitting color defects could be related to the small lateral size of hBN nanosheets dropped on SiO_2_, Si, and Al in comparison with larger-area-exfoliated hBN crystals with defect localization mainly at the edges and grain boundaries.^54^

We also studied the PL lineshape properties of many hBN nanosheets as a function of different underlying substrates. We measured the statistical sample of PL spectral properties. Deconvolution of each PL spectrum of a color center detected in hBN nanosheets has been performed by assuming Lorentzian lineshapes.^30^ As a result of deconvolution processes, until four peaks have been detected. Each PL spectrum was fitted with Lorentzian profiles to take into account the ZPL and SB emission (for more details, see discussion of Figure 7a,b). Particularly, we extracted the zero-phonon linewidth representing the ZPL peak full width at half-maximum (fwhm), and the ZPL/SB integrated intensity ratio for each used substrate. By taking into account the average values (for more details, see Discussions section), the ZPL fwhm shows an average value of around 27 meV for all used substrates, except thin Au. In this last case, we observed ZPL fwhm broadening up to an average value of 66 meV, indicating a significant decrease in emission quality. The mean ZPL/SB ratio determines the balance between ZPL emission and unwanted SB contribution in each PL spectra. It was found to be similar for Si, SiO_2_, and Al substrate (≈20–25%), and in accordance with ZPL fwhm analysis minimal for a thin Au substrate with an increase in the SB contribution with ZPL/SB ratio reduction to only 5%. From this analysis, it follows that PL emission distribution from few-layer hBN nanosheets is not substrate-dependent, except for a thin Au substrate, where PL quenching is observed and significant reduction of light emission properties.

**Figure 5:**
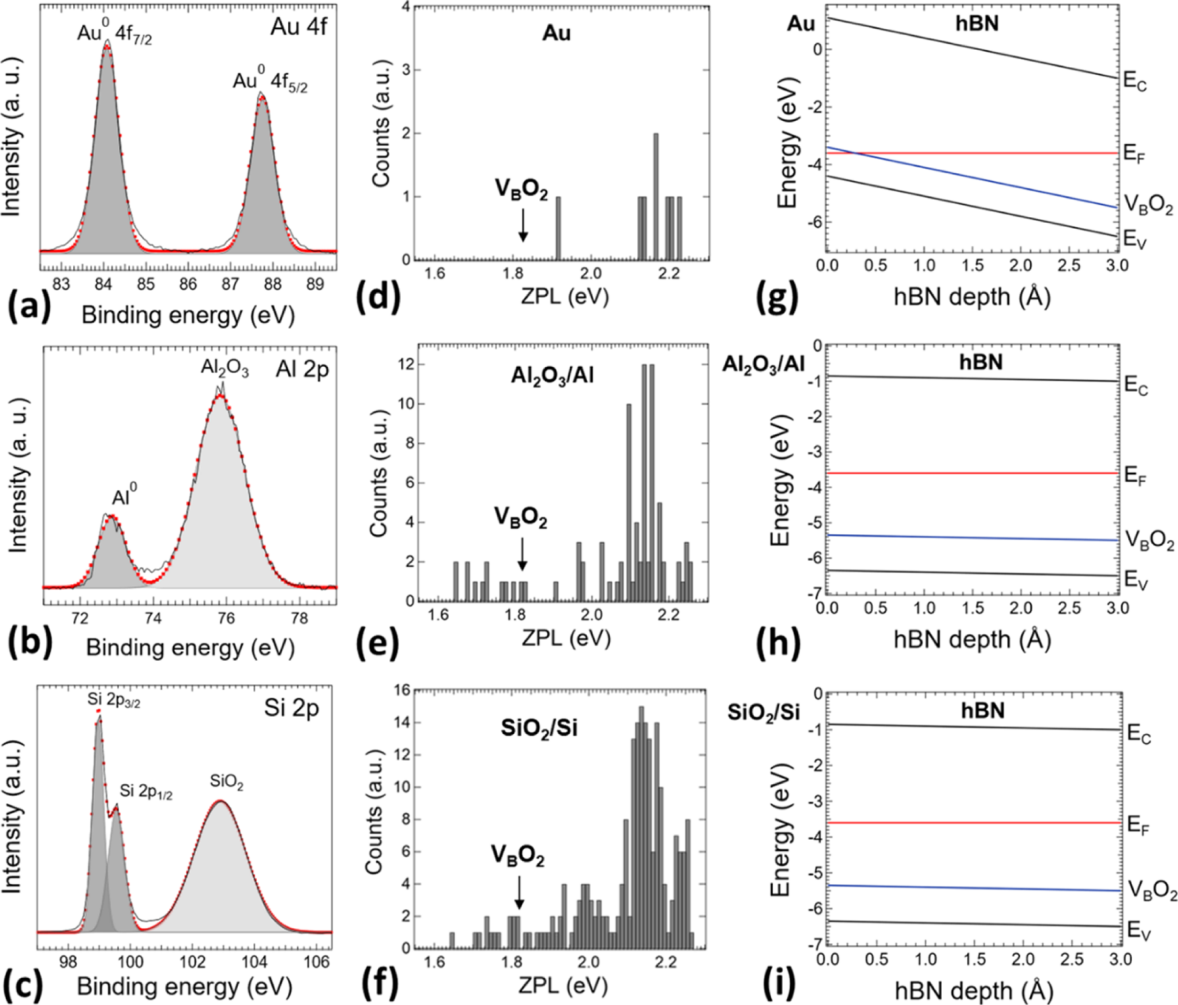
Comparison of light emission from color point defects in hBN nanosheets deposited on metallic Au, Al, and dielectric Si substrates. High-energy resolution XPS spectra of (a) Au 4f measured on the bare Au substrate, (b) Al 2p measured on the bare Al substrate, and (c) Si 2p measured on the Si substrate. Red cycles represent fitted values into the individual components. The surface of the Au substrate is pure metallic. On the other hand, the surface of the Al and Au substrate is oxidized by the Al_2_O_3_ and SiO_2_ layer, respectively. Histograms of zero-phonon line (ZPL) transition energy emission from point defects in hBN nanosheets deposited on (d) Au and oxidized (e) Al and (f) Si substrates. There is marked B-vacancy defect saturated with two oxygen atoms (V_B_O_2_) with ZPL transition at 1.85 eV^9^ as a possible candidate for emission at lower energies. Band-bending profile simulations with respect to the depth of the hBN monolayer from the interface with (g) Au and oxidized (h) Al and (i) Si substrates with shown transition energy of the V_B_O_2_ defect.^9^

It is well-known that PL quenching can occur when light-emitting 2D materials are in close contact with thin metal surfaces enabling a charge transfer between both materials.^55−59^ Among the different substrates employed here to study the optical properties of color centers in hBN, a mention apart deserves the case of Au. PL quenching was found only on the metallic Au substrate. In contrast to this, the Al substrate shows similar a PL behavior as dielectric/semiconducting substrates SiO_2_ and Si. One would say that the surface of the Al substrate might be oxidized by the used ozone treatment and thus not affecting the PL emission. To answer this, we applied X-ray photoelectron spectroscopy (XPS) chemical surface analysis of metallic Au, Al, and also semiconducting Si bare substrates (see Figure 5a–c). High-energy resolution XPS spectra of Au 4f are depicted in Figure 5a. The surface of the Au substrate is pure metallic. On the other hand, the surface of Al (Al 2p) and Si (Si 2p) substrate is oxidized even before an applied ozone treatment. In the case of the Al and Si substrate, a considerable amount of oxidized Al_2_O_3_ (Figure 5b) and SiO_2_ (Figure 5c) layer is present, respectively. It is clearly seen from the chemical analysis that only the Au substrate is pure metallic and thus enabling an electron transfer from localized centers in hBN nanosheets into the Fermi sea in Au. Histograms of ZPL transition energy emission from point defects in hBN nanosheets dispersed on Au, oxidized Al, and oxidized Si substrates are shown in Figure 5d–f and S3 in Supporting Information. Oxidized Al and Si substrates show a similar ZPL transition energy distribution of color defects from 1.65 to 2.7 eV, whereas the Au substrate causes quenching of ZPL transition energies lower than 1.9 eV. The origin of a lower energy emission is unknown. However, a B-vacancy defect saturated with two oxygen atoms (V_B_O_2_) with ZPL transition energy at 1.85 eV might be a possible candidate.^9^ We performed a band-bending profile simulation of an hBN monolayer with respect to its depth and interface with Au, oxidized Al, and oxidized Si substrates (Figures 5g–i S). A transition energy of the V_B_O_2_ defect^9^ is also shown there for the illustration. The Fermi level is positioned above the V_B_O_2_ defect transition energy in whole depth of the hBN monolayer deposited on oxidized Al and Si substrates. On the other hand, in the case of the Au substrate, the Fermi level is positioned below the V_B_O_2_ defect in close interface proximity with the Au substrate, resulting in quenching of the V_B_O_2_ and other color defects emitting in lower energy through nonradiative electron transfer processes. It follows that the nature of the defects created in hBN nanosheets might be located on the surface.

### 3.4 Preferential Surface Localization of Light Emitters in hBN Nanosheets

We continue our work with a study of atomic localization of color defects within the crystal lattice. Here, we pretend to clarify if the color defects are preferably located at the surface proximity or within an entire crystal volume. To unravel this question, we have investigated the PL emission as a function of hBN thickness ranging from 1 to 5 monolayers. Our sample thickness was identified by Raman spectroscopy due to the high frequency in-plane E_2g_ phonon peak shift, with a similar dependence as was observed for larger-area hBN flakes^3,36,61^ (see Figure S2 in Supporting Information) and with layer sensitivity up to 5 monolayers. We used Raman mapping to localize the position of hBN nanosheets with the corresponding thickness, and simultaneously, we recorded the corresponding PL spectra.

Figure 4d shows typical PL spectra for hBN nanosheets with thickness varying from 1 to 5 monolayers, showing a similar PL distribution including ZPL emission at around 2.16 eV and phonon SB. The probability to find an emitter acquired by counting hBN nanosheets having a Raman signal and at the same time with and without PL emission is depicted in Figure 4e. All measured hBN thicknesses exhibit a relatively high emitter probability around ≈30%. In fact, contrary to the literature,^46^ where fewer point color defect emitters were detected in single monolayer nanosheets in comparison with thicker hBN crystals, we found in monolayer a comparable number of emitters to thicker crystals. This indicates a reduced presence of surface charges and defects affecting phonon interactions^29^ that we attribute to the enhanced quality of hBN nanosheets and the preparation technique of samples used here. The observed high improvement of PL emission after surface ozone treatment (Figure 2d), PL emission quenching in Au interface proximity (Figure 5d,g), and a similar amount of light-emitting point defects in monolayer to thicker crystals (Figure 4e) indicate that point defects are localized preferentially on the hBN surface.

In order to investigate the characteristics of emitters in hBN nanosheets by varying thickness, we also analyzed their PL response by Lorentzian profile fittings (ZPL and SB contribution) (see Figure 7a,b). Figure 4f shows the results for ZPL fwhm and the ZPL/SB intensity ratio as a function of the hBN thickness. The ZPL/SB ratio (≈20%) and the ZPL fwhm (≈29 meV) for the hBN monolayer are comparable to that found in thicker crystals and especially fwhm is much narrower than values previously reported in ref (19), which also points out to an improvement in the structural quality of hBN nanosheets present in our samples.

### 3.5 Far-Field Emission Analysis as a Function of Substrate Material and hBN Thickness

To deeply understand the propagation of emitted light from hBN nanosheets, far-field angle-dependent emission patterns for the four different substrates and various hBN thickness (1–5 monolayers) were calculated (see Supporting Information for more detail) and compared with experimental results. In-plane emission was applied assuming that the dipole of the light emitter lies in the plane of the hBN monolayer.^30,32,33^ The radiating molecules within the 2D materials can be modeled as classical forced electric dipole oscillators distributed in the active layer.^62,63^ Such dipoles can have different orientations and emission frequencies and incoherently contribute to the far-field emission pattern. The electromagnetic waves of the dipoles propagated through the structure are influenced by the multiple reflections of different layers of the structure, complicating the optical modeling. Practically, a large number of dipoles need to be used to precisely compute the spatial emission pattern in such a method, which consumes extensive computational resources. The main idea of the reciprocity theorem is to convert a light out-coupling problem of a structure into a light in-coupling problem.^64−66^ It significantly simplifies the simulation and provides the information of the spatial emission pattern in a computationally efficient calculation.

**Figure 6:**
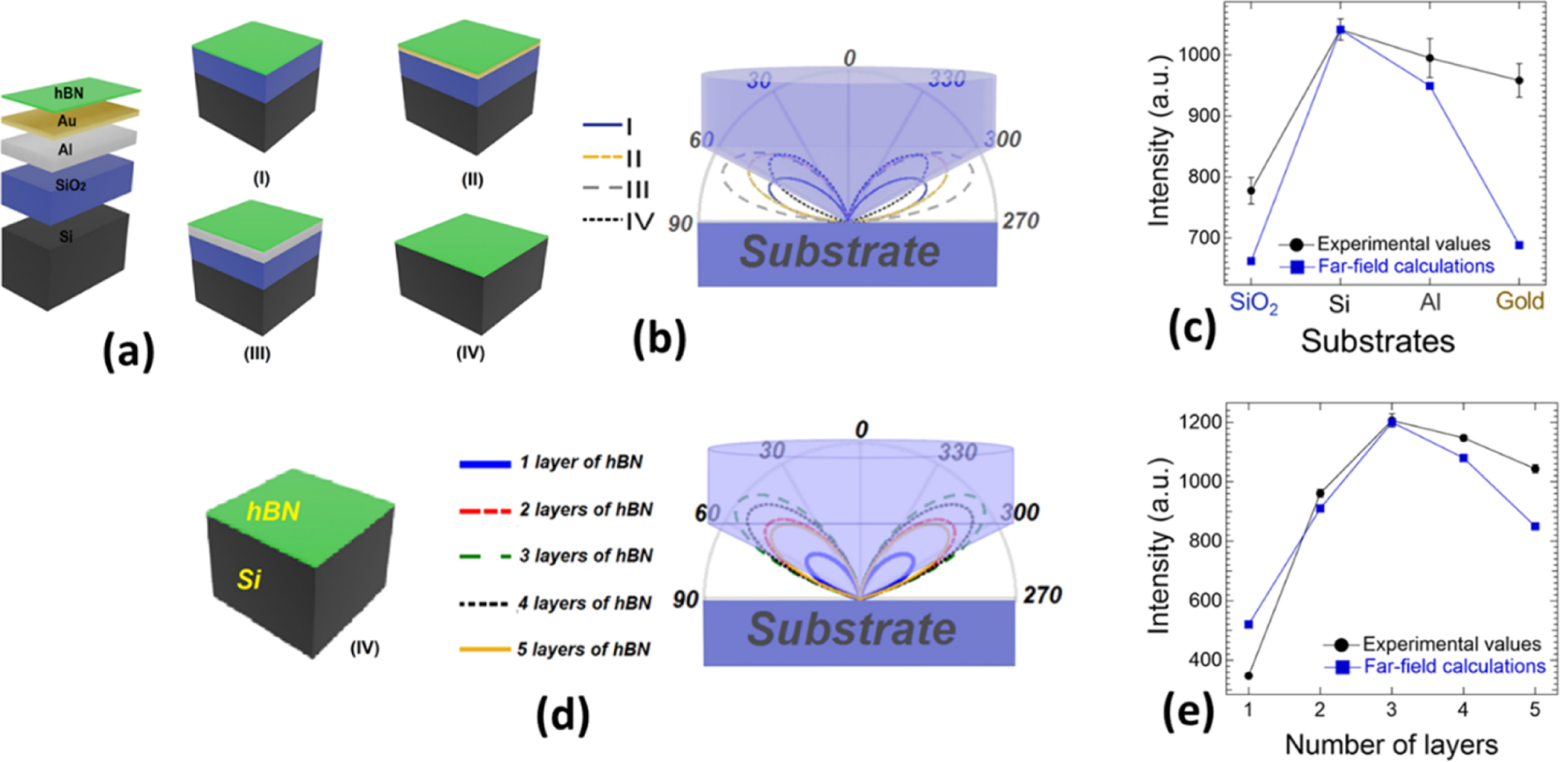
(a) Schematic representation of the structure of different substrates used for hBN exfoliation: (I) SiO_2_/Si with an oxidized layer thickness of 285 nm, (II) Au/SiO_2_/Si with a metal thickness of 30 nm, (III) Al/SiO_2_/Si with a metal thickness of 100 nm, and (IV) Si with a thickness of 380 μm. (b) Angular distribution of the radiated intensity from hBN nanosheets in the far field corresponding to the represented structures in (a). (d) Angular distribution of the radiated intensity in the far field as a function of number of hBN layers deposited on the substrate (IV). Here, as schematically shown in (b) and (d), the numerical aperture of the collecting lens is NA = 0.9 and the radiated patterns are calculated at λ = 575 nm (maximal emitted ZPL transition energy). Illustration of the maximum values of the radiated intensity as a function of (c) different substrates and (e) number of hBN layers for calculated and experimental values. The far-field values were normalized to the experimental values for the Si substrate with maximal intensity.

Figure 6a represents schematically four different substrates used in our experiments including (I) SiO_2_/Si (blue line), (II) Au/SiO_2_/Si (yellow line), (III) Al/SiO_2_/Si (gray line), and (IV) Si (black line). Oxidized surface layers on Al and Si substrates observed by XPS analysis (see Figure 5) were not included in the simulations because the influence of the thin (typically around 1 nm thick) oxidized layer on the far field will almost certainly be negligible when compared to a semi-infinite Al and Si substrate. Also, the refractive index of the lossless oxidized layers (SiO_2_ and Al_2_O_3_) is much smaller than the bare ones (Si and Al) and thus insignificant on the far field. Analysis of the far-field emission patterns of the substrates is illustrated in Figure 6b. Angular distribution of the radiated intensity in the far field and angular reflection separately for each of the substrates in Si is presented in Figure S6. The emission patterns were calculated for an emission wavelength of 575 nm (≈2.16 eV), which is maximal detected ZPL transition energy (see Figure 3d). All substrates have similar symmetric ellipsoidal-like shapes with a higher PL emission intensity on metallic substrates than on dielectric ones. However, a very important factor affecting an amount of collected emission intensity is the numerical aperture of the objective, which should be considered. In our experiments 0.9 NA objective has been used. For illustration, in Figure 6b, the spot size of the collected light emission is highlighted. As a result, the maximal collection has been found for the substrate (IV) with almost all radiated intensity located at the collection lens area. On the other hand, emission losses caused by the maximal emission angle located out of the collecting lens area have been found for the rest of the substrates. The calculated collection efficiency is shown in Figure 6c and compared with the ZPL intensity experimental results as a function of the substrate material. In agreement with the experimental results, the maximal collected PL emission is found on the substrate (IV) and the lowest on substrate (I). Calculated PL mission from metallic substrates is also consistent with the experimental results with a higher collected PL intensity for the substrate (III) than the substrate (II).

We have calculated the far field of the in-plane light emission as a function of emission angle and the hBN layer thickness. Figure 6d shows the angular distribution of the radiated intensity in the far field calculated as a function of the number of hBN layers located on top of the Si substrate (for the angular distribution on the SiO_2_/Si substrate, see Figure S7 in Supporting Information). We considered the different percentages of emitters experimentally found in hBN crystals by varying thickness from 1–5 monolayers in the calculations, where the maximum number of point defect emitters was found in samples with 3–4 monolayers. Specifically, these calculations are performed by exchanging the locations of the localized time-harmonic current density and the location of the field evaluation (more details in Supporting Information). In light of this, a different percentage of charges in hBN flakes is accounted for in the current density as different dipole moment strength, where the current density is assumed to be an oscillating dipole with the origin at the charge distribution’s center (center of each flake). Figure 6e shows a comparison of the maximum values of calculated radiated intensity and experimental values corresponding to the average intensity of ZPL emission versus number of the hBN layers for the Si substrate. Both theoretical and experimental values are in good agreement with each other, after normalizing the maximum emission intensity for three monolayers. Both theoretical and experimental values indicate that thicker hBN nanosheets, where point defect emitters might be localized farther from the substrate, exhibit a higher brightness compared with monolayers (see Figure 6e), which is in correspondence with results reported in the literature.^26^

## 4 Discussion

**Figure 7:**
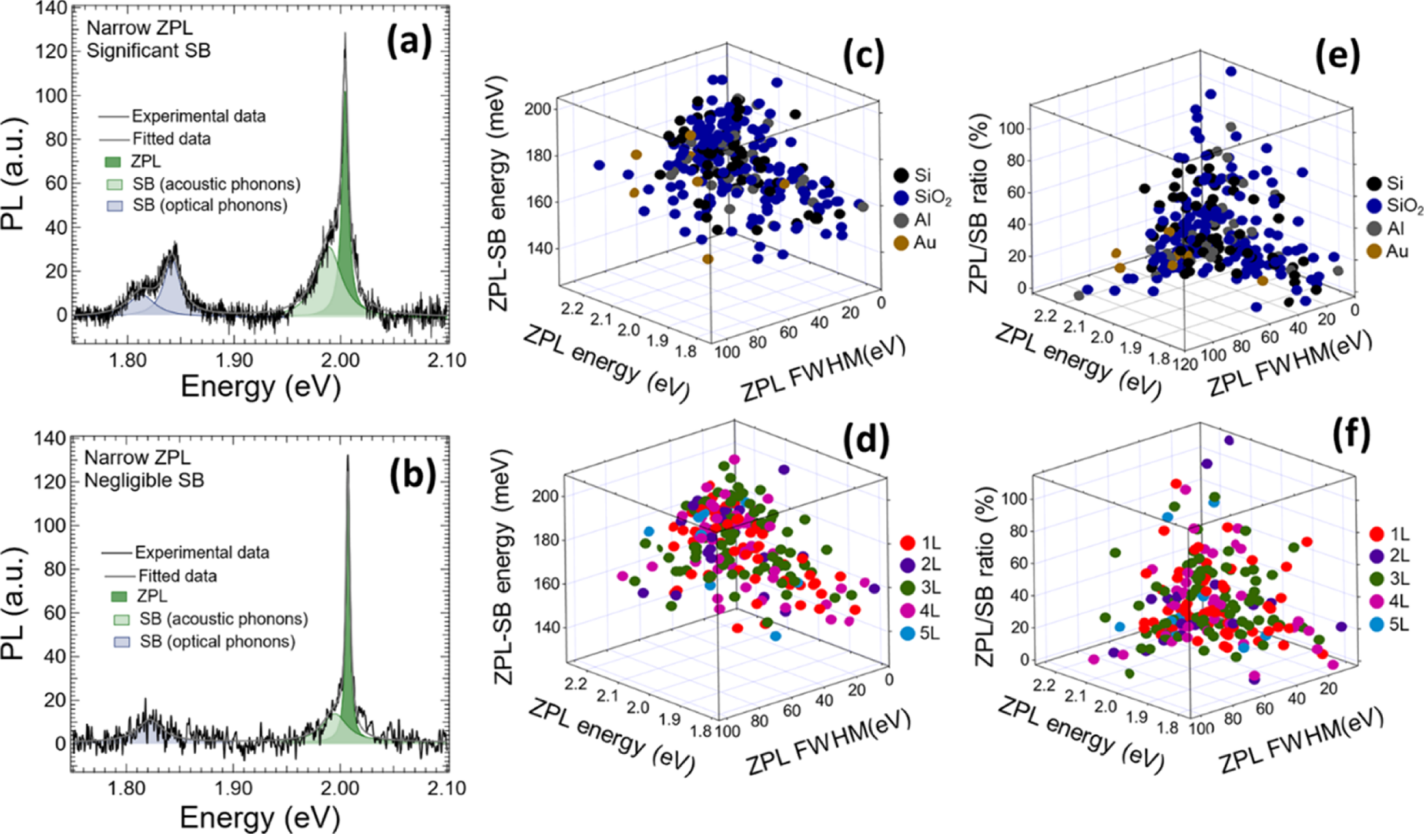
Photoluminescence (PL) spectra of two different emitters showing both very narrow zero-phonon line (ZPL) emission, differing by (a) significant and (b) negligible contribution of side-band (SB) emission. The ZPL and SB peaks were fitted to Lorentzian functions. The ZPL peak is attributed to transition energy of a point defect, a higher energy SB peak is assigned to longitudinal acoustic phonons,^30^ and lower-energy SB peaks are belonging to longitudinal optical phonons.^30^ Statistics of many PL spectra were calculated by Lorentzian fitting of ZPL and SB emission. Three-dimensional visualization of ZPL and SB energy difference as a function of ZPL fwhm and ZPL transition energy for (c) different substrates and (d) hBN thickness. Three-dimensional visualization of the ZPL/SB integrated intensity ratio as a function of ZPL fwhm and ZPL transition energy for (e) different substrates and (f) hBN-nanosheet thickness.

From our results, it follows that the structural quality of hBN nanosheets and surface is very important for the spectral features observed in light emission associated to point defects in hBN. As discussed above, this quality can be enhanced by the reduction of charge traps via thermal annealing and ozone treatment,^35^ as demonstrated in the present work for few-layer hBN nanosheets. To understand more deeply a quality of PL emission, that is, distribution of ZPL and SB emission, we performed statistics of many color defects in hBN nanosheets (1–5 L) deposited on different substrates. As an example, Figure 7a,b shows the PL spectrum of two color centers detected in hBN nanosheets, where each one of the components resolved by deconvolution of the PL spectra have been included. The ZPL typically shows a peak asymmetry, which has been attributed to acoustic phonon interaction^30^ or to a second electronic transition.^67^ The PL spectra also typically exhibit significant SB emission^21,30^ separated from the ZPL by an energy shift of approximately 170 meV,^10,30,67^ attributable to optical phonon interactions^30^ or electron-phonon coupling.^21,68^ From the PL statistics carried out on many different point emitters, we can conclude that the ZPL-SB energy difference is roughly constant with an average value of around 170 meV for different substrates (Figure 7c) and hBN thicknesses (Figure 7d), in accordance with the literature.^10,67^ This observation means that point defects are mainly located in a given 2D-hBN monolayer and energetically are not affected by the number of monolayers of the investigated nanosheet nor by the chemical nature of their interface with the different substrates. However, the ZPL-SB energy difference exhibits a certain variation with the ZPL energy and a noticeable dispersion. In the first case, one would say that the ZPL-SB energy difference would have minimum values of around 150 meV for *E*_ZPL_ ≈1.85 eV that would correspond to point defects V_B_O_2_–Si-1–O_B_O_B_V_N_–V_B_Si_i_V_N_^9,15^ (see Figure 3d). In the second case, a high dispersion of the ZPL-SB energy difference is observed from 120 to 200 meV, approximately, which would be indicative of a different origin of point defects (V_N_C_B_, V_N_N_B_, C_B_N_B_V_N_, O_N_,..) giving rise to more probable ZPL energies of 2.10–2.25 eV^13,15^ (see Figure 3d) or their energy changes and phonon coupling by lattice deformations.^32,49^

To evaluate the quality of the optical emission, we also estimated the ZPL fwhm and ratio between ZPL and SB integrated intensities (ZPL/SB ratio) for all substrates and hBN thicknesses. Figure 7c–f shows statistics of the ZPL fwhm as a function of the ZPL energy. The ZPL fwhm values range as much as from ≈2 to 117 meV for different substrates (Figure 7c–e) and hBN thicknesses (Figure 7d–f). In accordance with the ZPL-SB statistics, we have found a higher dispersion for ZPL peak energies between 2.10 and 2.25 eV. These variations should be related to the enhanced electron–phonon interaction of the different point defects and charge traps emitting at higher energies. This trend is in good correspondence with previous work reported in the literature.^30^Figure 7e,f shows the dependence of the ZPL fwhm as a function of the ZPL/SB ratio, with a similar trend for all used substrates (Figure 7e) and hBN nanosheet thickness (Figure 7f). Increase in the ZPL/SB ratio (lowering contribution of SB emission) leads to narrowing of the ZPL fwhm due to a weaker coupling to longitudinal optical phonons or reduced charge traps^30,35^ and thus enhancement of the light emission properties. PL spectra of two different emitter characteristics with very narrow ZPL emission are depicted in Figure 7a,b. However, some differences between the both spectra can be found. The first emitter (see Figure 7a) is affected by a significant contribution of unwanted SB emission, which reduces its optical quality. On the other hand, the second emitter (see Figure 7b) shows just a negligible SB emission. These high-quality emitters with a very narrow ZPL linewidth and negligible SB emission could be very interesting candidates for several photonic applications.

Despite the similar emission properties on Si, SiO_2_, and Al substrates, a discrepancy has been found on the Au substrate (see Figure 4). We have measured strong PL quenching (Figure 4b) together with a higher occurrence of the SB emission and broadening of the ZPL fwhm (see Figure 4c), resulting in a stronger electron–phonon interactions and higher nonradiative energy transitions.

With a focus on emission properties as a function of hBN thickness, the monolayer shows a comparable ZPL fwhm to thicker crystals (see Figure 4f) indicating its higher crystal quality and lower presence of surface charge traps. On the other hand, thicker crystals than just a one monolayer show a higher brightness (see Figures 6e and S7b in Supporting Information) and a smaller contribution from the SB (Figure 4f). Hence, assuming the emitter localization on the hBN surface, the light emission from thicker hBN is not energetically affected by the interlayer or substrate interactions, which could result in a stronger phonon coupling. It is clear that the modulation of the electron and photon interaction as a function of energy, that is, as a function of the type of defect/trap, should be treated with great attention when using hBN and its interfaces to develop stable, efficient, and high single-photon purity emitters. Therefore, considerable attention should be required to prepare high-structural quality hBN crystals, an appropriate surface post-treatment, and a suitable choice of interfaces from both sides of the crystal lattice to maximize the optical quality of these point color defects.

## 5 Conclusions

In summary, the present investigation has demonstrated the possibility to define single-photon emitters based on point defects in few-layer hBN with the processing method described here (heated substrate and ozone treatment), the light emission properties being optimum (theory and experiment) when nanosheets are deposited on silicon substrates. Moreover, it is possible to find and study single emitters originated in point defects of different chemical origins throughout the whole visible wavelength range with very narrow ZPL (several meV) and/or low/negligible SB contribution at room temperature. In addition, single defects emitting at lower energies than 1.9 eV can be extrinsically quenched by the gold substrate. These results are relevant for the deterministic generation of color defects in hBN nanosheets. Moreover, the simplicity of the methods proposed here to successfully generate room-temperature emitters in hBN nanosheets at the same time that the structural quality of the nanosheet improves is important for future applications in quantum photonics.

## Supporting Information Available

Scheme of the hBN hexagonal honey-comb lattice structure of double-layer; optical microscopy image of hBN nanosheets exfoliated on the Si substrate at high and low coverage; SEM image of hBN nanosheets exfoliated on the Si substrate at high and low coverage; histogram of the Raman peak central position with values corresponding to the hBN thickness from 1 to 5 monolayers; Raman spectra of hBN thickness varying from 1 to 5 monolayers; average Raman peak high and Raman peak fwhm width as a function hBN thickness from 1 to 5 monolayers; histograms of zero-phonon line (ZPL) transition energy emission of point defects in hBN nanosheets deposited on SiO_2_ substrates; histograms of zero-phonon line (ZPL) transition energy emission of point defects in hBN nanosheets with different thicknesses deposited on the SiO_2_/Si substrate: 1 layer, 2 layers, 3 layers, 4 layers, and 5 layers; three-dimensional visualization of statistics of ZPL fwhm for different substrates and hBN thickness as a function of ZPL and SB energy difference and SB/ZPL integrated intensity ratio; three-dimensional visualization of statistics of ZPL transition energy for different substrates and hBN thickness as a function of ZPL and SB energy difference and SB/ZPL integrated intensity ratio; angular distribution of the radiated intensity in the far field of hBN on top of different substrates including (I) SiO_2_/Si (blue line), (II) Au/SiO_2_/Si (yellow line), (III) Al/SiO_2_/Si (gray line), and (IV) Si (black line); angular reflectance spectra of different structures corresponding to (a) which is calculated at λ = 575 nm; schematic image of the SiO_2_/Si substrate covered by hBN and the angular distribution of the radiated intensity in the far field as a function of the hBN layers, here, the numerical aperture of the collecting lens is NA = 0.9 and the radiated patterns are calculated at λ = 575 nm (maximal emitted ZPL transition energy); and illustration of the maximum values of the radiated intensity versus the number of the hBN layers for calculated and experimental values (PDF)
