# Supplementary material for: Extrinsic Effects on the Optical Properties of Surface Color Defects Generated in Hexagonal Boron Nitride Nanosheets
Source: ACS Appl Mater Interfaces. 2021 Sep 14;13(38):46105–16. doi: 10.1021/acsami.1c11060 (PMC8485329; doi:10.1021/acsami.1c11060)
Supplement: Supplementary file 1 — am1c11060_si_001.pdf [file am1c11060_si_001.pdf]

Supporting information

# Extrinsic Effects on the Optical Properties of Surface Colour Defects generated in Hexagonal Boron Nitride Nanosheets

Marie Krečmarová[1], Rodolfo Canet-Albiach[1], Hamid Pashaei-Adl[1], Setatira Gorji[1], Guillermo Muñoz-Matutano[1], Miloš Nesládek[2], Juan P. Martínez-Pastor[1] and Juan F. Sánchez-Royo[1*]

[1]Instituto de Ciencia de Materiales, Universidad de Valencia (ICMUV), P.O. Box 22085, 46071 Valencia, Spain

[2]Institute for Materials Research, Material Physics Division University of Hasselt, Wetenschapspark 1, B 3590 Diepenbeek, Belgium

*****Correspondence: Juan.F.Sanchez@uv.es

### Preparation of light emitting hBN nanosheets

The hBN is an electrical insulator with a large band gap. A schematic of atomic lattice structure of hBN double-layer is shown in Figure S1a. It consists of boron (blue) and nitrogen (red) atoms arranged like graphene in a hexagonal honey-comb lattice structure with individual layers coupled by van der Waals forces. Each boron atom is covalently bonded through $sp^2$ hybridization to three neighbouring nitrogen atoms with a bond length of approximately 1.45 Å. Individual layers of hBN lattice are coupled by weak van der Waals forces with an interlayer spacing of 0.333 nm. Values of crystal spacing were taken from [1].

Few-layer hBN nanosheets purchased from commercially available pristine hBN from Graphene Supermarket were dispersed on different substrates. Thickness of the nanosheets vary from 1 to 5 monolayers and their lateral size from 50 - 200 nm. Figure S1 shows optical microscopy and scanning electron microscopy (SEM) images of dispersed hBN nanosheets on Si substrate in areas of high (S1b-c) and low (S1d-e) hBN nanosheets coverage. We used the high hBN substrate coverage for investigation of optical properties of emitters, i.e. photoluminescence (PL) and Raman mapping.

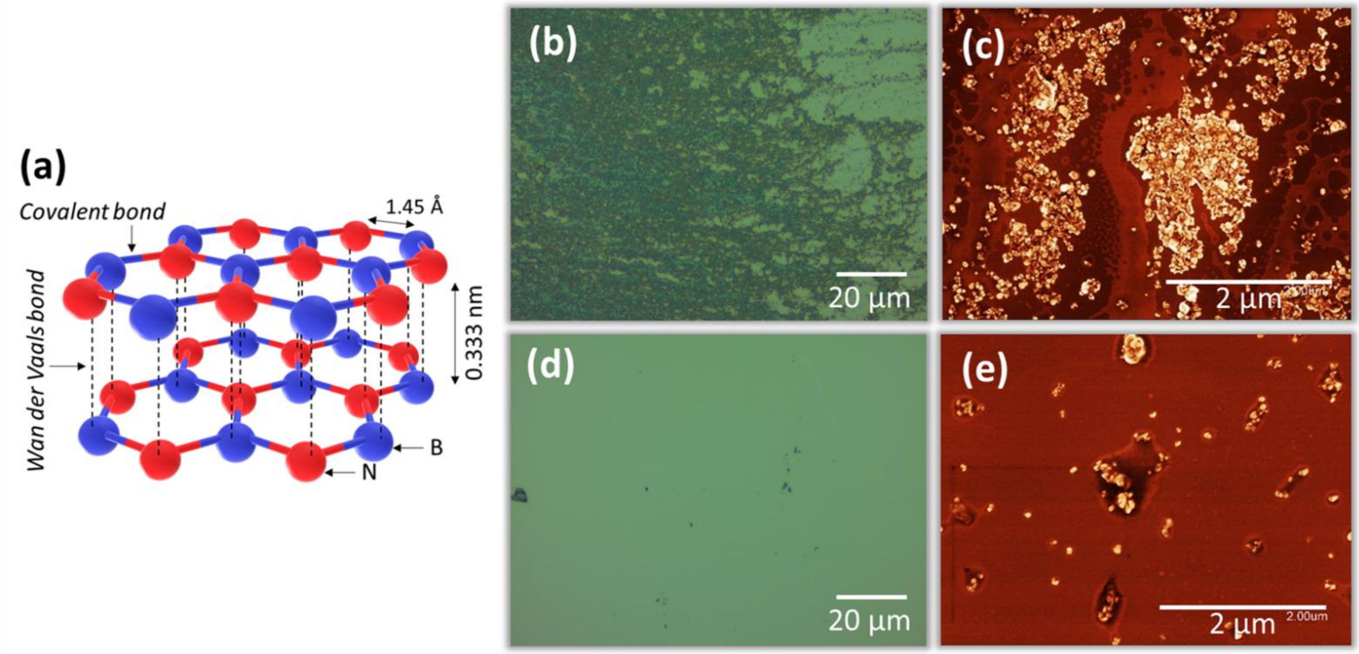


**Figure S1.** (a) Scheme of the hBN hexagonal honey-comb lattice structure of double-layer with blue and red colour circles corresponding to boron and nitrogen atoms, respectively. Optical microscopy image of hBN nanosheets deposited on Si substrate at (b) high and (d) low coverage concentration. Scanning electron microscopy (SEM) image of hBN nanosheets deposited on Si substrate at (c) high and (e) low coverage concentration.

## Micro-photoluminescence and micro-Raman spectroscopy analysis

Then we focused on investigation of PL emission as a function of the hBN thickness ranging from 1 to 5 monolayers and different underlying substrates. The thickness of hBN was indentified from the energy shift of the high frequency in-plane $E_{2g}$ phonon peaks up to 5 monolayers similary as was reported for larger area flakes [2, 3, 4] by micro-Raman spectroscopy with excitation wavelength at 532 nm. The histogram of measured Raman peak position for hBN nanosheets with thicknesses from 1 to 5 monolayers is depicted in Figure S2a. By taking into account a phonon centre position maxima with comparison to our previous research [2] and literature [3, 4], we have identified corresponding phonon peak positions with average values at 1369.7 $cm^{-1}$ for 1 layer, 1369.1 $cm^{-1}$ for 2 layers, 1367.4 $cm^{-1}$ for 3 layers, 1366.8 $cm^{-1}$ for 4 layers and 1365.7 $cm^{-1}$ for 5 layers (see Figure S2b). Raman spectra of hBN nanosheets with thicknesses ranging from 1 to 5 monolayers are shown in Figure S2c. We have found and increasing intensity of the Raman peak for thicker crystals (Figure S2d) and almost unchanged full width at half maximum (FWHM) peak width for all thicknesses (Figure S2e).

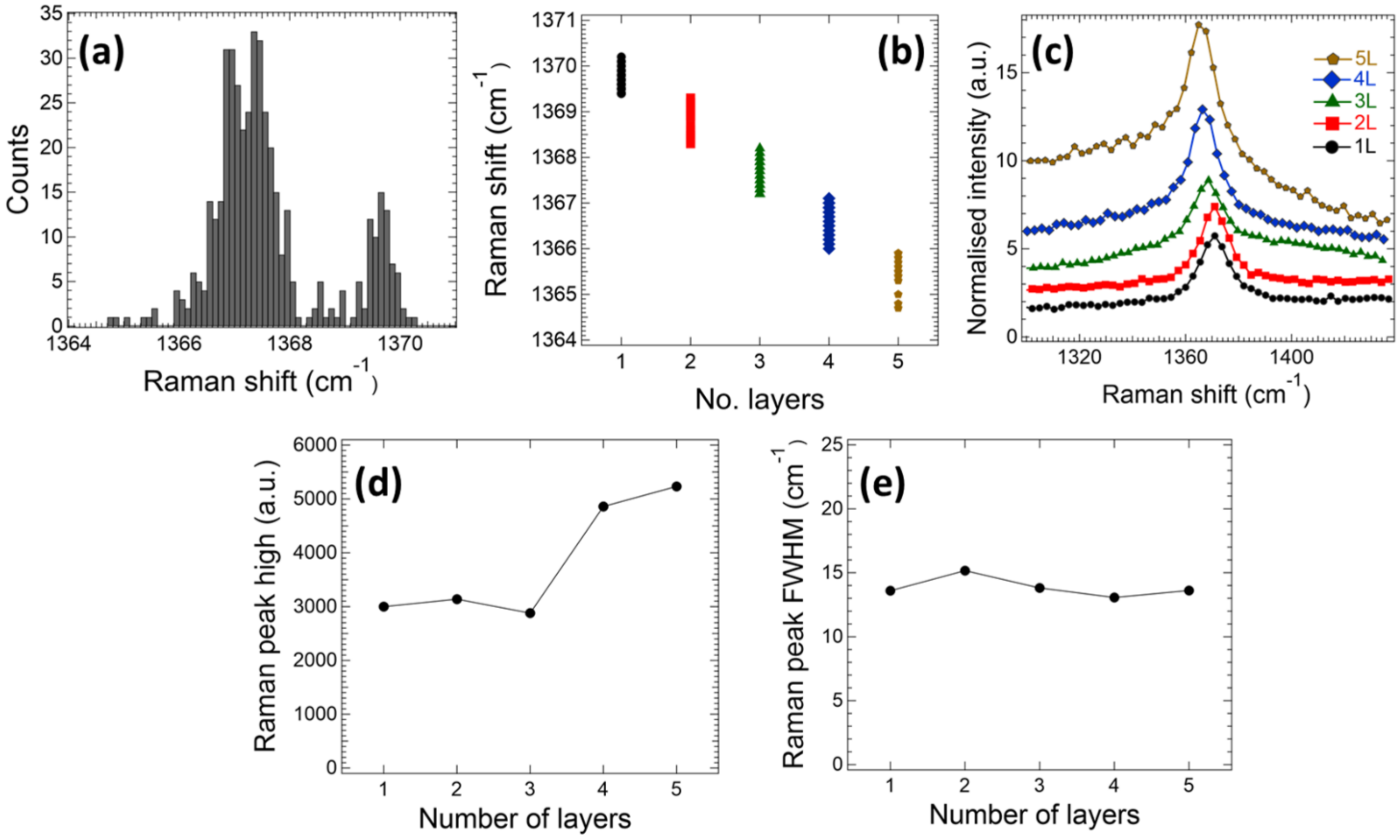


**Figure S2.** (a) Histogram of Raman peak central position with (b) values corresponding to hBN thickness from 1 to 5 monolayers. (c) Raman spectra of hBN thickness varying from 1 to 5 monolayers. Average (d) Raman peak high and (e) Raman peak full width at half maximum (FWHM) width as function hBN thickness from 1 to 5 monolayers.

Figure S3 shows a histogram of zero-phonon line (ZPL) transition energies over the visible spectrum for $SiO_2$ substrate with a broad emission range distribution from 1.97 to 2.27 eV and a maximal ZPL central energy located at 2.16 eV. We also studied light emission as function of hBN thickness. Histograms of ZPL emission for thickness from 1 to 5 monolayer are presented in Figure S4a-e. The distribution is similar for all samples thicknesses except 5 monolayers, where only few nanosheets were measured. The maximal ZPL transition emission energy maximum was found consistently around 2.16 eV for all measured hBN nanosheets thicknesses.

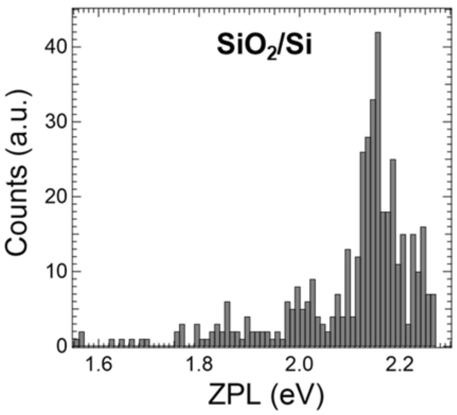


**Figure S3.** Histograms of zero-phonon line (ZPL) transition energy emission of point defects in hBN nanosheets deposited on $SiO_2$/Si substrates.

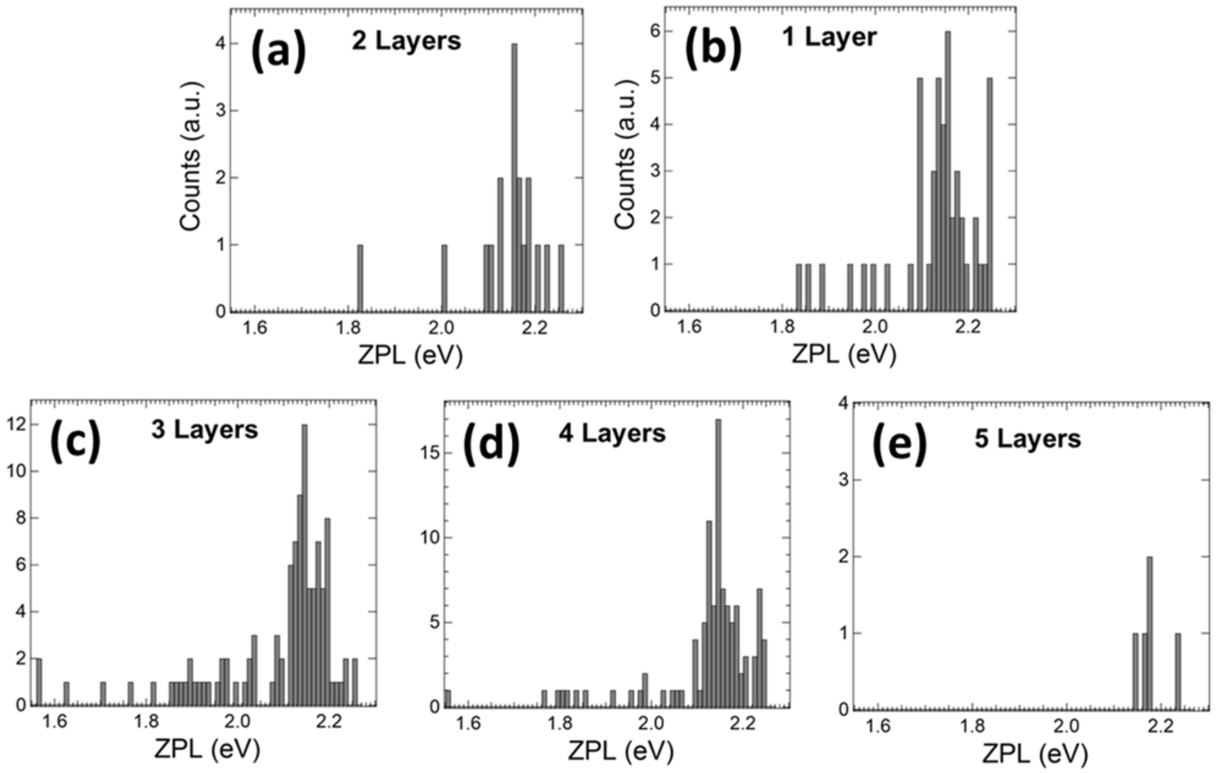


**Figure S4.** Histograms of zero-phonon line (ZPL) transition energy emission of point defects in hBN nanosheets with different thickness deposited on $SiO_2$/Si substrate: (a) 1 layer, (b) 2 layers, (c) 3 layers, (d) 4 layers and (e) 5 layers.

Then we used ZPL and side band (SB) peak position, FWHM and integrated intensity as fitting parameters for PL statistics along the entire range of ZPL transition energies in order to investigate light emission properties. We calculated ZPL FWHM as function of ZPL/SB integrated intensities ratio and ZPL- SB energy difference for all substrates (Figure 5a) and hBN thickness (Figure 5c) with a similar trend. The maximal ZPL FWHM and SB/ZPL ratio is dispersed over the ZPL-SB energy differences (from 120 to 200 meV) with the highest incidence around 170 meV corresponding to the average value of average ZPL-SB energy difference reported in the literature [56] and also found by us. We also calculated ZPL energy dependence as function ZPL/SB integrated intensities ratio and ZPL-SB energy difference for all substrates (Figure 5b) and hBN thickness (Figure 5d) with a similar trend. ZPL transition energy with the SB/ZPL variation as much as from almost 1 % to 100 % suggesting different quality of the light emission. There is visible a similar trend as for ZPL FWHM and peak intensity with a higher SB/ZPL ratio dispersion for higher ZPL transition energies, which could be related to the enhanced electron-phonon interaction of the different single defects and charge

traps emitting at higher energies. This trend is in good correspondence with previous works reported in the literature [7].

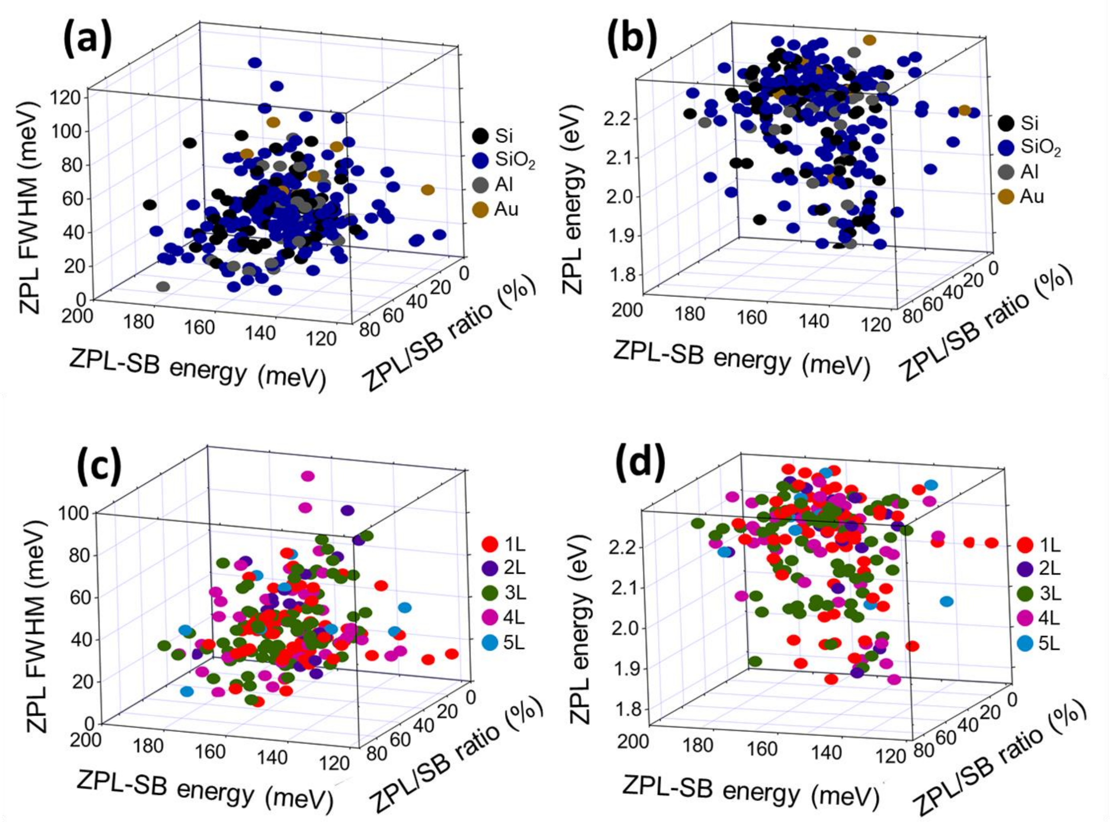


**Figure S5.** Three-dimensional visualization of statistics of zero-phonon line (ZPL) full width at half maximum (FWHM) for (a) different substrates and (c) hBN thickness as function of ZPL and side-bands (SB) energy difference, and SB/ZPL integrated intensity ratio. Three-dimensional visualization of statistics of ZPL transition energy for (b) different substrates and (d) hBN thickness as function of ZPL and SB energy difference, and SB/ZPL integrated intensity ratio.

## Far-field calculations

According to the Lorentz reciprocity theorem [8, 9, 10], exchanging the locations of the current and the location where the field is evaluated have no impact on the relationship between a localized oscillating current and the resultant electric field. Accordingly, this theorem is a powerful technique for far-field evaluation in various science territories related to the point sources [8, 9, 10].

$$\iiint \vec{E}_{J_1}.\vec{J}_2\, d^3\vec{r} = \iiint \vec{E}_{J_2}.\vec{J}_1\, d^3\vec{r} \tag{1}$$

Where $\vec{J}$ is the localized time-harmonic current density oscillating at an angular frequency of $\omega$ and $\vec{E}$ is the consequential electric field resulted from the current density. Here it is worthy to mention that, this theorem is valid for absorbing and anisotropic media but not for nonlinear media.

Moreover, the angular reflectance spectra of the aforementioned structures have been calculated using well-known transfer matrix methods (TMM). The transfer matrix can be achieved directly using the boundary condition [11, 12] of the electromagnetic field at the interference of the adjacent layers. The electric and magnetic field at the two positions z and z + Δz in the same layer can be related via the following transfer matrix [13, 14]:

$$M_l(\Delta z) = \begin{pmatrix} cos(k_z\Delta z) & -\frac{\mu\omega}{k_z c} sin(k_z\Delta z) \\ \frac{k_z c}{\mu\omega} sin(k_z\Delta z) & cos(k_z\Delta z) \end{pmatrix} \tag{2}$$

Here, *l*=1, 2… N, where N is the total number of the layers in the structure.

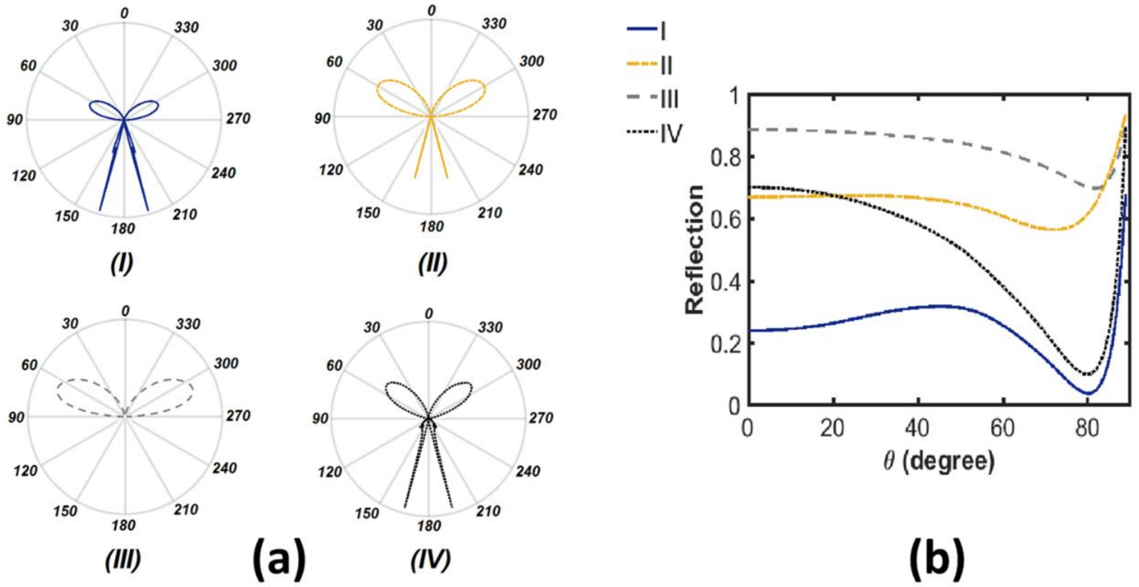


**Figure S6.** (a) the angular distribution of the radiated intensity in the far-field of hBN on top of different substrates including (I) $SiO_2$/Si (blue line), (II) Au/$SiO_2$/Si (yellow line), (III) Al/$SiO_2$/Si (grey line) and (IV) Si (black line). (b) Angular reflectance spectra of different structures corresponding to (a) which calculated at λ=575 nm.

Figure S6a shows the angular distribution of the radiated intensity in the far-field separately for each of the substrates. Moreover, by using the transfer matrix method the angular reflection of the substrates has been calculated (Figure S6b). We can understand some of these behaviours using basic assumptions. Substrate (I) should have minimum intensity because not only some part of the light penetrates to the substrate but also this substrate has minimum reflection. Substrate (II) should have lower field intensity than the substrate (III), due to the lower reflection but also due to the propagation of apportion of light through the thinner metal layer. A noticeable result of this comparison is related to the substrate (IV): although the reflection and far-field intensity of this substrate are not as high as substrate (II) and substrate (III), its maximum takes place for smaller angles, which means that most of the emitted light will be collected with the objective lens in our experiment. As a result, the more intense signal is obtained using substrate (IV: Si), since the main part of the emitted light is within the numerical aperture of the objective

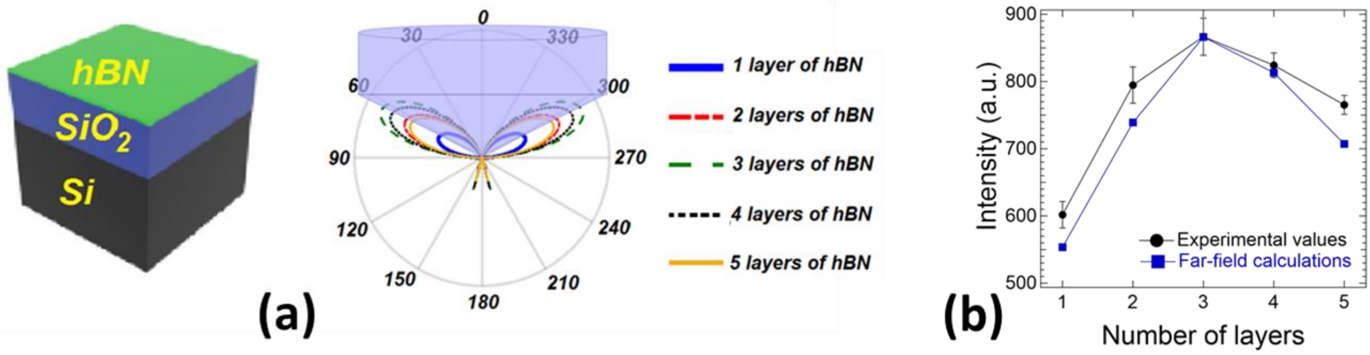


**Figure S7.** (a) Schematic image of $SiO_2$/Si substrate covered by hBN and the angular distribution of the radiated intensity in the far-field as a function of the hBN layers. Here the numerical aperture of the collecting lens is NA=0.9 and the radiated patterns are calculated at λ=575 nm (maximal emitted ZPL transition energy). (b) Illustrate the maximum values of the radiated intensity versus the number of the hBN layers for calculated and experimental values. The Far-field values were normalised to the experimental values for Si substrate with maximal intensity.

Figures S7a shows the angular distribution of the radiated intensity in the far-field calculated as a function of the number of hBN layers on $SiO_2$/Si substrate. These calculations are done by considering the different percentage of emitters experimentally found in hBN crystals by varying thickness from 1-5 monolayers,

where the maximum number of point defect emitters was found in samples with 3-4 monolayers. Concretelly, calculations are performed by exchanging the locations of the localized time-harmonic current density and the location of the field evaluation. The relationship between the localized time-harmonic current density generated by a point source and the dipole moment is given by $\vec{J}_m = -i\omega\vec{P}_m\delta(\vec{r} - \vec{r}_m)$, where $\vec{P}_m$ (m=1 or 2) is the dipole moment then considering equation 1 $\vec{E}_{P1}.\vec{P}_2 = \vec{E}_{P2}.\vec{P}_1$.

Figure S7b shows a comparison of the maximum values of calculated radiated intensity and experimental values corresponding to the average intensity of ZPL emission versus number of the hBN layers. Both theoretical and experimental values are in good agreement with each other, after normalizing the maximum emission intensity for 3 monolayers. In accordance with results reported in the previous paragraphs, higher emission intensity was found on Si substrate than on $SiO_2$/Si substrate.

# References


S(1) Wang, J.; Ma, F.; Sun, M. Graphene, Hexagonal Boron Nitride, and Their Heterostructures: Properties and Applications. *RSC Adv.* **2017**, *7* (27), 16801–16822. https://doi.org/10.1039/C7RA00260B.

S(2) Krečmarová, M.; Andres-Penares, D.; Fekete, L.; Ashcheulov, P.; Molina-Sánchez, A.; Canet-Albiach, R.; Gregora, I.; Mortet, V.; Martínez-Pastor, J. P.; Sánchez-Royo, J. F. Optical Contrast and Raman Spectroscopy Techniques Applied to Few-Layer 2D Hexagonal Boron Nitride. *Nanomaterials* **2019**, *9* (7), 1047. https://doi.org/10.3390/nano9071047.

S(3) Li, L. H.; Cervenka, J.; Watanabe, K.; Taniguchi, T.; Chen, Y. Strong Oxidation Resistance of Atomically Thin Boron Nitride Nanosheets. *ACS Nano* **2014**, *8* (2), 1457–1462. https://doi.org/10.1021/nn500059s.

S(4) Cai, Q.; Scullion, D.; Falin, A.; Watanabe, K.; Taniguchi, T.; Chen, Y.; Santos, E. J. G.; Li, L. H. Raman Signature and Phonon Dispersion of Atomically Thin Boron Nitride. *Nanoscale* **2017**, *9* (9), 3059–3067. https://doi.org/10.1039/C6NR09312D.

S(5) Martínez, L. J.; Pelini, T.; Waselowski, V.; Maze, J. R.; Gil, B.; Cassabois, G.; Jacques, V. Efficient Single Photon Emission from a High-Purity Hexagonal Boron Nitride Crystal. *Phys. Rev. B* **2016**, *94* (12), 121405. https://doi.org/10.1103/PhysRevB.94.121405.

S(6) Bommer, A.; Becher, C. New Insights into Nonclassical Light Emission from Defects in Multi-Layer Hexagonal Boron Nitride. *Nanophotonics* **2019**, *8* (11), 2041–2048. https://doi.org/10.1515/nanoph-2019-0123.

S(7) Wigger, D.; Schmidt, R.; Del Pozo-Zamudio, O.; Preuß, J. A.; Tonndorf, P.; Schneider, R.; Steeger, P.; Kern, J.; Khodaei, Y.; Sperling, J.; de Vasconcellos, S. M.; Bratschitsch, R.; Kuhn, T. Phonon-Assisted Emission and Absorption of Individual Color Centers in Hexagonal Boron Nitride. *2D Mater.* **2019**, *6* (3), 035006. https://doi.org/10.1088/2053-1583/ab1188.

S(8) Yang, J.; Hugonin, J.-P.; Lalanne, P. Near-to-Far Field Transformations for Radiative and Guided Waves. *ACS Photonics* **2016**, *3* (3), 395–402. https://doi.org/10.1021/acsphotonics.5b00559.

S(9) Zhang, S.; Martins, E. R.; Diyaf, A. G.; Wilson, J. I. B.; Turnbull, G. A.; Samuel, I. D. W. Calculation of the Emission Power Distribution of Microstructured OLEDs Using the Reciprocity Theorem. *Synthetic Metals* **2015**, *205*, 127–133. https://doi.org/10.1016/j.synthmet.2015.03.035.

S(10) Zhang, P.; Ren, P.-L.; Chen, X.-W. On the Emission Pattern of Nanoscopic Emitters in Planar Anisotropic Matrix and Nanoantenna Structures. *Nanoscale* **2019**, *11* (23), 11195–11201. https://doi.org/10.1039/C9NR00235A.

S(11) Entezar, S. R.; Habil Karimi, M.; Adl, H. P. Optical Isolation via One-Dimensional Magneto-Photonic Crystals Containing Nonlinear Defect Layer. *Optics Communications* **2015**, *352*, 91–95. https://doi.org/10.1016/j.optcom.2015.04.071.

S(12) Pashaeiadl, H.; Naserpour, M.; Zapata-Rodríguez, C. J. Scattering of Electromagnetic Waves by a Graphene-Coated Thin Cylinder of Left-Handed Metamaterial. *Optik* **2018**, *159*, 123–132. https://doi.org/10.1016/j.ijleo.2018.01.056.

S(13) Pashaei Adl, H.; Bayat, F.; Ghorani, N.; Ahmadi-Kandjani, S.; Tajalli, H. A Defective 1-D Photonic Crystal-Based Chemical Sensor in Total Internal Reflection Geometry. *IEEE Sensors Journal* **2017**, *17* (13), 4046–4051. https://doi.org/10.1109/JSEN.2017.2701845.

S(14) Yeh, P. *Optical Waves in Layered Media*, 1. Edition February 2005.; Wiley Series in Pure and Applied Optics (Series Nr. 1); Wiley & Sons Ltd.
